# Preparation of 4-Amino-3-hydrazino-1,2,4-triazol-5-thiol-Modified Graphene Oxide and Its Greatly Enhanced Selective Adsorption of Gallium in Aqueous Solution

**DOI:** 10.3390/molecules29122778

**Published:** 2024-06-11

**Authors:** Xi Zhu, Yong Guo, Baozhan Zheng

**Affiliations:** College of Chemistry, Sichuan University, Chengdu 610065, China; 265cici@163.com (X.Z.); zhengbaozhan@scu.edu.cn (B.Z.)

**Keywords:** graphene oxide, nitrogen heterocycle, covalent modification, recovery, selective adsorption, trivalent gallium

## Abstract

Efficient recovery of gallium (Ga) from vanadium slag processing residue (VSPR) solution is of great significance for environmental protection and resource utilization, but improving its selective adsorption against the coexisting Sc^3+^ and In^3+^ is still challenging. Herein, a novel adsorbent consisting of 4-amino-3-hydrazino-1,2,4-triazol-5-thiol (AHTZT)-modified graphene oxide (GO-AHTZT) was successfully synthesized that exhibits a higher adsorption selectivity for Ga^3+^ in VSPR solution with coexisting Sc^3+^ and In^3+^. Under optimal conditions, the adsorption capacity of GO-AHTZT for Ga^3+^ can reach 23.92 mg g^−1^, which is 4.9 and 12.6 times higher than that for Sc^3+^ (4.87 mg g^−1^) and In^3+^ (1.90 mg g^−1^) adsorption, indicating the excellent anti-interference ability of GO-AHTZT against Sc^3+^ and In^3+^. The process and mechanism of Ga^3+^ adsorption onto GO-AHTZT was also studied and discussed in detail. By measuring the adsorption process and by characterizing the adsorbent before and after adsorption, we demonstrate that the selective interaction between the Ga^3+^- and N-containing groups in AHTZT is the main reason for the improved adsorption selectivity. This work opens up an avenue for the design and synthesis of highly selective adsorbents for Ga^3+^ in complex VSPR solutions.

## 1. Introduction

The extraction of vanadium from vanadium titano-magnetite ores by salt roasting is one of the most representative routes to produce vanadium products. After sodium vanadate is leached out, other metals, including indium (In), gallium (Ga) and scandium (Sc), at low concentration levels remain in the vanadium slag processing residue (VSPR), causing human health risks, environmental pollution, and wastage of resources [1]. Relatively little information can be retrieved from the literature regarding the adsorption/enrichment of Ga, In and Sc in aqueous solutions, especially with specific reference to practical applications [2]. More importantly, one of these metals, Ga, has been widely used for the preparation of novel semiconductor materials. For example, gallium arsenide is one of the most promising semiconductor materials for manufacturing optoelectronics [3], and gallium arsenic phosphorus and gallium aluminum arsenic can be utilized as solid excitation materials for optical fiber communication systems, solar cells and large-scale high-speed integrated circuits [4]. Therefore, the enrichment and recovery of Ga from VSPR has highlighted the importance of environmental protection and resource utilization.

The extraction of Ga from the residues produced during the process metallurgy of aluminum, electrolytic zinc, fly ash and other sources dominates its production [5]. The recovery of Ga is mainly carried out through acid–base leaching and further isolation–purification, and the latter process includes precipitation, extraction, ion exchange, supported liquid membranes (SLMs) and chlorination [6]. However, these recovery processes suffer from many disadvantages, including low total efficiency, high energy consumption and too many impure components. Therefore, the development of more efficient and lower energy consumption processes for Ga recovery is becoming a hot topic. The recovery of Ga from zinc residues by pyrometallurgical and hydrometallurgical methods has also been successfully implemented [7,8]. However, some limitations, such as the co-extraction of other impurities and the utilization of difficult-to-separate extractants, have apparently become an obstacle for further applications [6].

Recently, the separation of metal ions from aqueous solutions by adsorption has attracted great attention due to its high selectivity, high efficiency, easy-to-operate process and cost-saving properties [9,10]. The efficient and precise separation of Ga by adsorption has become a hot research topic; undoubtedly, adsorbents may play an important role in the process. For example, based on the interaction between the carboxyl groups and Ga^3+^, 90% of the Ga^3+^ can be adsorbed from acidic solutions by several chelating adsorbents, such as 3,4-dihydroxybenzoic acid and 3,4,5-trihydroxybenzoic acid functionalized pre-aminated polymeric resins [11]. However, the adsorption selectivity of these chelating adsorbents needs to be improved because the interactions between the carboxyl groups and other coexisting metal ions cannot be ignored. In comparison, chitosan (CS)-derived porous carbon that was further activated by KOH at 800 °C (CS-800) exhibited good adsorption selectivity for Ga^3+^ rather than for other metal ions such as Zn^2+^, Cu^2+^, Ge^4+^ and Al^3+^ [12]. Although the detailed adsorption mechanism was not further discussed by the authors, we propose that the nitrogen (N)-containing functional groups might contribute to the adsorption. Further research studies are moving forward. The hydrothermal carbonization of urea and discarded persimmon and ZnCl_2_ pyrolysis could produce a novel biomass carbon aerogel with high adsorption selectivity for Ga^3+^, confirming that the abundant nitrogen (N)-containing functional groups contribute a lot to adsorbent–adsorbate interactions [13].

Besides these classic and regular adsorbents, nanocarbon-based materials are increasingly being used as efficient adsorbents for the recovery of various metal ions [14]. Graphene oxide (GO), which possesses abundant oxygen-containing functional groups such as C-O-C, C=O and -COOH, can be used as an excellent adsorbent. GO can be further functionalized with other materials to produce well-controlled, versatile composites with special adsorption characteristics [15]. The adsorption capacity of GO for Al^3+^ and Ga^3+^ was investigated, and its affinity for Ga^3+^ could be observed [14]; that is, the oxygen-containing functional groups are favorable for adsorption, which could be further proven from the adsorption of Ga^3+^ by persimmon tannin/GO composites [16] and polyacrylic acid-functionalized GO [17]. However, the adsorption selectivity of these oxygen-containing ligand-modified GO compounds still needs to be improved. Interestingly, N-containing ligands have exhibited better selective interactions with specific metal ions such as Au^3+^, Cu^2+^ or Cd^2+^ to achieve selective adsorption [18,19,20], and 4-amino-5-mercapto-1,2,4-triazole as an efficient flotation reagent might prefer to adsorb Cu^2+^ rather than Fe^2+^ [21]. Although the selective adsorption of Ga^3+^ by N-containing ligand-based adsorbents has rarely been studied, previously reported research has also indicated that N-containing ligand-based complexes possessed higher multi-responsive activities toward Ga^3+^ rather than In^3+^ [22]. Another investigation has compared the complexing capacity of 4-amino-3-thio-1,2,4-triazole, 4-amino-3-thio-5-methyl-1,2,4-triazole and 4-amino-6-methyl-3-thio-1,2,4-triazin-5-one toward Ga^3+^ and Co^3+^, again confirming the higher affinity of 1,2,4-triazole-based ligands for Ga^3+^ [23]. As discussed above, the utilization of N-containing ligands for the construction of GO-based adsorbents could produce novel adsorbents with excellent adsorption selectivity for Ga^3+^.

It is worth noting that the rational selection of novel 1,2,4-triazole-based ligands with specific functional groups that can covalently bond with GO is important and essential. Herein, to construct a novel GO-based adsorbent for Ga^3+^ recovery, we tried to develop a 4-amino-3-hydrazino-1,2,4-triazol-5-thiol (AHTZT) covalently functionalized GO (GO-AHTZT) composite. The adsorption properties of the GO-AHTZT composite toward Ga^3+^ were investigated in detail. The morphology, composition and chemical state of the GO-AHTZT composite pre- and post-adsorption Ga^3+^ were characterized by various methodologies such as scanning electron microscopy (SEM) equipped with X-ray energy dispersive spectroscopy (EDS), elemental mapping analysis, X-ray photoelectron spectroscopy (XPS), thermographic analysis (TGA) and Fourier transform infrared (FT-IR) spectroscopy. An adsorption mechanism was also proposed. Interestingly, the adsorption of Ga^3+^ by the developed GO-AHTZT composite is highly pH dependent, which is beneficial for the recovery of the adsorbent after adsorption.

## 2. Results and Discussion

### 2.1. Characterizations

**Scheme 1:**
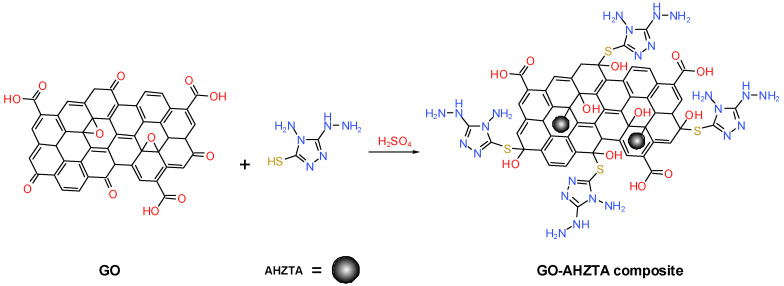
A schematic diagram for the preparation of the GO-AHZTA composite.

The thiol group (-SH) has generally been considered one of the most reactive nucleophiles [24,25]. We propose that the amino and hydrazine groups might be protonated under acidic conditions, leaving the active thiol group of AHZTA to react with the carbonyl (C=O) and epoxy groups of GO to produce a GO-AHZTA composite (Scheme 1). Therefore, the surface of the GO-based composite might be covered by AHZTA, thus providing specific active sites to interact with rare scattering elements and guaranteeing selective adsorption. To determine the structure, morphology, composition, and chemical state of the GO-AHZTA composite, further characterizations such as SEM, EDS, elemental mapping, XPS, FT-IR and TGA were carried out.

#### 2.1.1. SEM, EDS, and Elemental Mapping Analyses

**Figure 1:**
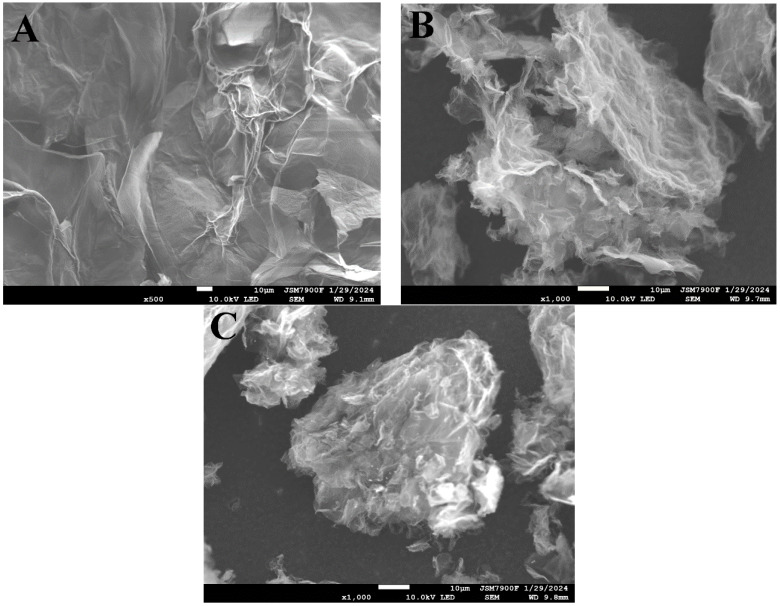
SEM images of the samples: (**A**) GO; (**B**) GO-AHZTA composite; (**C**) GO-AHZTA composite post-Ga^3+^ adsorption.

Figure 1 shows the morphologies of the GO and GO-AHZTA composites pre- and post-adsorption of Ga^3+^ as detected by SEM. As exhibited in Figure 1A, GO possesses typical lamellar, flexible sheet-like structures with irregular wrinkles, confirming that the successful exfoliation of flake graphite by violent oxidation occurred. We can clearly observe that the wrinkles near the edges of the GO sheets are aligned with the contact lines of neighboring sheets; the formation of such basic microstructures would affect the physicochemical properties, including electrical, optical, adsorptive and functionalization characteristics [26]. The chemical grafting of AHZTA will endow the GO-AHZTA composite with abundant surface-active sites to selectively interact with specific molecules, and the thickened structures due to the stacking interactions will further guarantee that the exposure of other sites can be effectively avoided (Figure 1B). The GO-AHZTA composite post-Ga^3+^ adsorption ^+^ remains almost unchanged, retaining the sheet-like, stacking structures, suggesting that the newly developed adsorbent can maintain structural stability during the oscillating adsorption process (Figure 1C).

**Figure 2:**
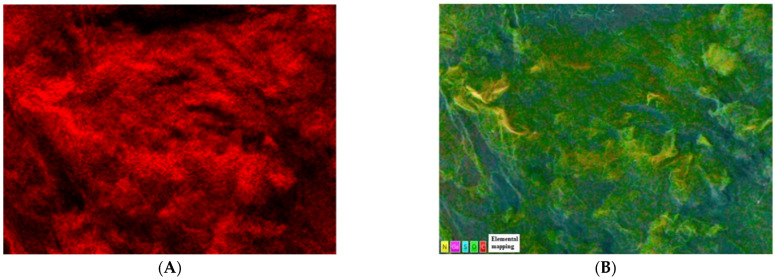

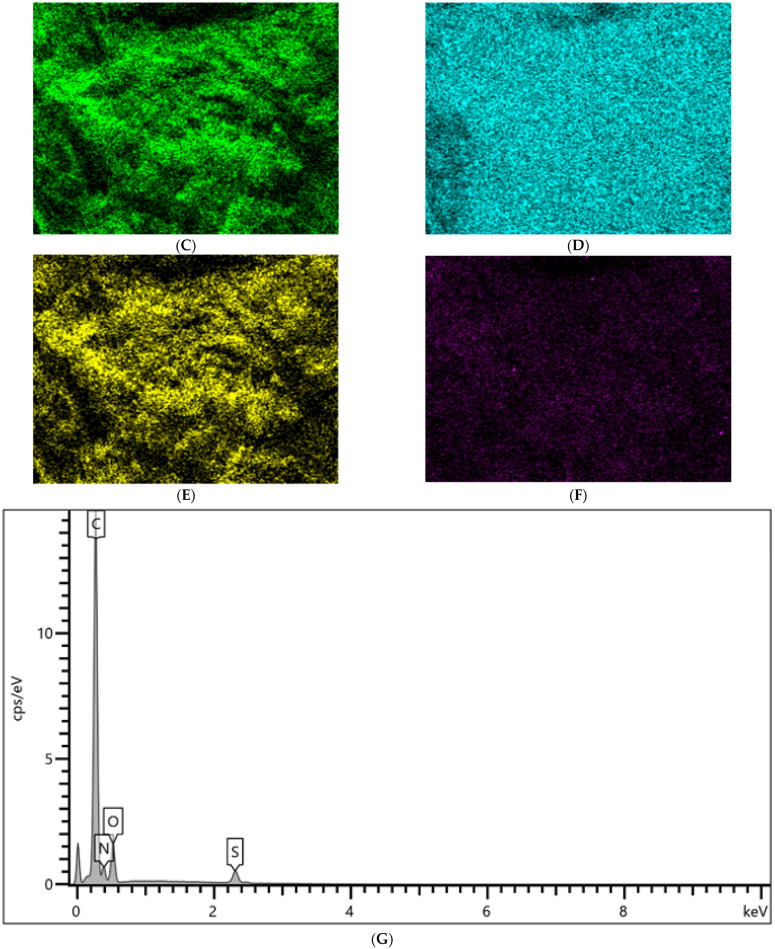
Elemental mapping images of the GO-AHZTA composite post- Ga^3+^ adsorption: (**A**) Area tested; (**B**) C; (**C**) O; (**D**) S; (**E**) N and (**F**) Ga^3+^. (**G**) EDS of the GO-AHZTA composite post- Ga^3+^ adsorption.

To reveal the elemental (C, O, S and N) distributions in the GO-AHZTA composite and the relationship with Ga^3+^, the adsorbent post-adsorption is further investigated by elemental mapping analyses (Figure 2A–F). Obviously, the surface of the sample is almost fully covered by element S, suggesting that covalent coupling of AHZTA onto GO occurs (Figure 2D). In addition, the uniform distribution of element N along with elements C and O could be confirmed (Figure 2B,C,E). The Ga^3+^ distribution is more consistent with element N, suggesting that the adsorption might occur at active N-containing sites due to strong cation-lone pair interactions (Figure 2C,F) [27,28], which we later confirmed by XPS. The existence of around 65.66 wt.% of C, 9.51 wt.% of O, 9.03 wt.% of S, 15.54 wt.% of N and 0.26 wt.% of Ga^3+^ in the GO-AHZTA composite post- Ga^3+^ adsorption was also determined by EDS, indicating that not all of element N participates in the adsorption process (Figure 2G).

#### 2.1.2. FT-IR Spectra

**Figure 3:**
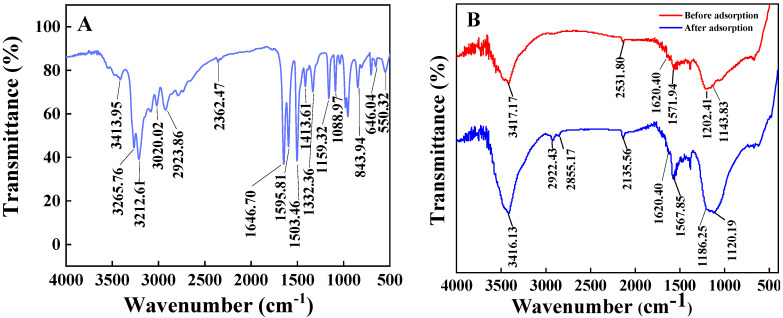
FT-IR spectra of the samples: (**A**) AHZTA and (**B**) GO-AHZTA composites pre- and post-Ga^3+^ adsorption.

The surface functional groups of the samples (AHZTA and GO-AHZTA composites pre- and post- Ga^3+^ adsorption) were investigated by FT-IR spectroscopy (Figure 3). For AHZTA (Figure 3A), the characteristic peaks observed at wavenumbers 3413.95, 3265.76 and 3212.61 cm^−1^ correspond to the stretching vibrations of the associated hydrogen bonds and amino (-NH) group [29]. The weak characteristic peak observed at 2362.47 cm^−1^ could be attributed to the stretching vibration of the mercapto group (S-H). The peak at wavenumber 1646.70 cm^−1^ corresponds to the stretching vibration of the C=N bond. The peak at wavenumbers 1595.81 and 1503.46 cm^−1^ could be assigned to the skeletal vibrations of the aromatic heterocycle. The peak at wavenumber 843.94 cm^−1^ could be attributed to the stretching vibration of the C=S bond, indicating that a thione tautomer will be formed even in the solid state. The weak peaks at wavenumbers 550.32 and 646.04 cm^−1^ again confirm the existence of C-S bonds [30].

In comparison with the FT-IR of GO (Figure S1) [31] and AHZTA (Figure 3A), the GO-AHZTA composite (Figure 3B) possesses a weak peak corresponding to the C-S bond at wavenumber 672.58 cm^−1^, while the peaks corresponding to the C=S bond of the thione tautomer and the stretching vibration of the C=O groups of GO disappear, respectively, suggesting that AHZTA is chemically grafted onto GO through the nucleophilic addition of thiol groups. The disappearance of the characteristic S-H peak also suggests that the residual uncombined AHZTA is successfully removed from the composite by repeated rinsing, while a C-S bond is formed between GO and AHZTA by the nucleophilic addition to the carbonyl and epoxy groups (Scheme 1). The peak (1620.40 cm^−1^) corresponding to the C=O stretching vibrations of carboxyl groups remains almost unchanged, indicating that nucleophilic addition did not occur at the C=O of the -COOH groups, which is inconsistent with the reaction characteristics of carboxylic acids [32]. Additionally, the broad peaks corresponding to the associated hydrogen bonds of N-H and O-H (~3417.17 cm^−1^), the stretching vibration of the C=N bond (1571.94 cm^−1^), the skeleton vibrations of aromatic heterocycles (1620.40 cm^−1^), the peak of C-N stretching vibrations (1202.41 cm^−1^) and the peak of N-N stretching vibrations (1143.83 cm^−1^) could also be detected. After adsorbing Ga^3+^, the peaks corresponding to the C=N, C-N and N-N are slightly red-shifted to the lower wavenumbers of 1567.85, 1186.25 and 1120.19 cm^−1^, respectively, suggesting that the N-containing groups contribute to the effective absorption.

#### 2.1.3. XPS

**Figure 4:**
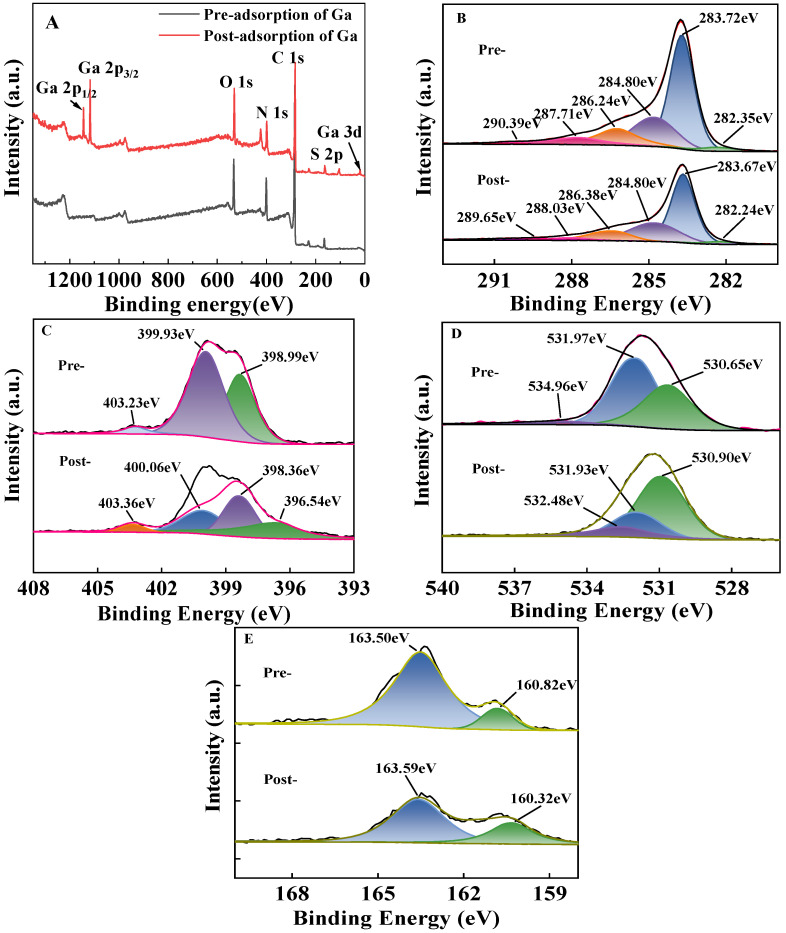
XPS of the GO-AHZTA composite pre- and post-Ga^3+^ adsorption: (**A**) The survey spectra; and XPS-peak-differentiation-imitating analyses of (**B**) C1s, (**C**) N1s; (**D**) O1s and (**E**) S2p.

To investigate the chemical state and composition of the GO-AHZTA composite, X-ray photoelectron spectroscopy (XPS) was conducted (Figure 4). The survey spectra exhibit the coexistence of C, O, N, S and Ga elements in the GO-AHZTA composite post-Ga^3+^ adsorption (Figure 4A). The two peaks with binding energies of 1118.2 and 1145.6 eV, corresponding to Ga 2p_3/2_ and Ga 2p_1/2_, respectively, confirm its highest chemical oxidation state (Ga^3+^) in the composite [33], which is further elucidated by the peak of Ga 3d_5/2_ at 22.3 eV.

XPS-peak-differentiation-imitating analyses of N 1s are shown in Figure 4C. The N 1S spectrum can be fitted into four peaks, which are attributed to positively charged N-H (403.36 eV) and –C=N (400.06 eV), N-C (398.36 eV) and N-N (396.54 eV), respectively [34]. The peaks slightly shift in comparison with those (403.23, 399.93 and 398.99 eV) of the pristine composite, again suggesting that coordination interactions between the N-containing groups of the GO-AHZTA composite and Ga^3+^ occur. 

The C1s spectrum of the GO-AHZTA composite post- Ga^3+^ adsorption can be deconvolved into six peaks located at 289.65, 288.03, 286.38, 284.8, 283.67 and 282.24 eV, which correspond to the O=COH, C=O, C-O, C=C, C-S and C=N bonds, respectively (Figure 4B) [35]. In comparison with the peaks in the original composite located at 290.39, 287.71, 286.24, 284.80, 283.72 and 282.35 eV, the binding energies shift to lower binding energies, confirming the intermolecular interactions between the GO-AHZTA composite and Ga^3+^.

The O1s XPS spectra of GO-AHZTA-Ga (Figure 4D) can be deconvolved into two peaks centered at about 532.48, 531.93 and 530.90 eV, which correspond to C-O-C, -O-C=O and O-H bonds, respectively [36,37]. The peak of the O-C=O bond is scarcely changed in comparison with that (531.97 eV) of pristine composite, confirming that the O-C=O bond is not involved in the adsorption. The peaks of the GO-AHZTA composite corresponding to C-O-C and O-H bonds at 534.96 and 530.65 eV are slightly changed, which are generally attributed to various kinds of -OH groups and chemisorbed oxygen and water [37,38]. 

The S2p peaks of GO-AHZTA-Ga at 160.32 eV and 163.59 eV correspond to the characteristic peaks of S2p_3/2_ and S2p_1/2_, respectively (Figure 4E) [39], which remain almost unchanged in comparison with those (160.82, 163.50 eV) of GO-AHZTA, indicating that S-containing groups are not involved in the adsorption process. Similarly, the binding energies of the deconvolved peaks corresponding to O1s, N1S and S2S are all shifted in comparison with those in the pristine composite (Figure 4C–E). The XPS results demonstrate the characteristic bands of C-S bonding and confirm the formation of the GO-AHZTA composite, as well as suggest the possible intermolecular interactions between the GO-AHZTA composite and Ga^3+^.

#### 2.1.4. TGA

**Figure 5:**
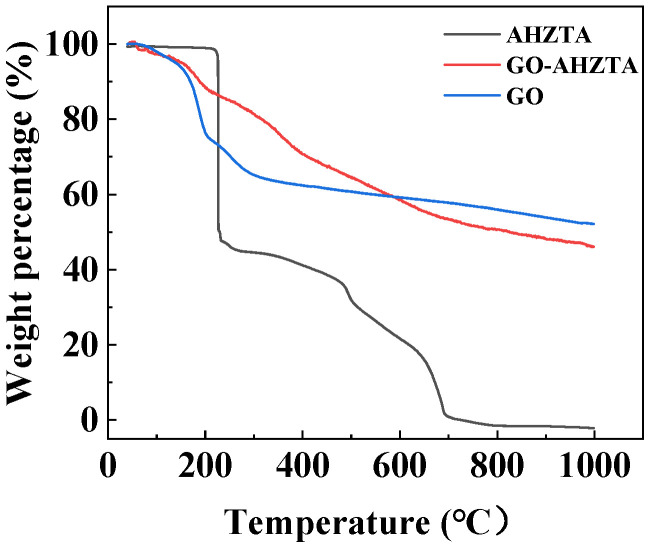
TGA curves of the AHZTA, GO and GO-AHZTA composites.

As shown Figure 5, three main temperature regions (TRs) with characteristic mass loss steps for AHZTA are revealed in the TGA curves: TR-I (200–245.5 °C), corresponding to the pyrosis of N-containing branched chains; TR-II (245.5–501.3 °C), attributed to the thermal cleavage of the thiol group; and TR-III (501.3–800.5 °C), related to carbon combustion. Excluding the effects of oxygen-containing groups of GO, around 26.52 wt.% of AHZTA is grafted onto GO [40]. It is worth noting that the GO-AHZTA composite exhibits a higher pyrolysis temperature than AHZTA in the test temperature range, confirming that covalent coupling between GO and AHZTA had occurred.

### 2.2. Adsorption Data

#### 2.2.1. Adsorption Selectivity

**Figure 6:**
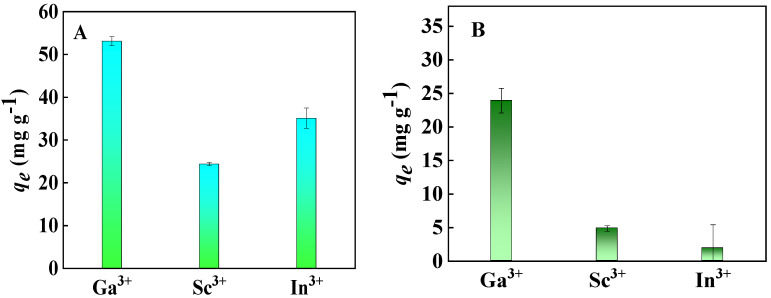
Adsorption capacities of the GO-based adsorbent for Ga^3+^, Sc^3+^ and In^3+^ (*C*_0_ = 50.0 mg L^−1^; *t* = 60 min; *T* = 298 K; dosage of adsorbent = 5.0 mg): (**A**) GO; (**B**) GO-AHTZT composite.

The control experiments using only GO were implemented, and the adsorption capacity (*q_e_)* of 53.12, 24.4 and 35.12 mg g^−1^ for rare scattering elements including Ga^3+^, Sc^3+^ and In^3+^ could be observed (Figure 6A). Obviously, the higher adsorption capacity and lower selectivity of GO could be attributed to the abundant -COOH groups on GO dominating the adsorption process. In addition, it was very difficult for the GO to be separated from the aqueous solutions after adsorption, which greatly limited its practical application. Therefore, various adsorbents including UiO-66 series metal–organic framework composites [41], persimmon tannin/GO composites [16] and polyacrylic acid functionalized GO [17] were rationally designed and utilized for the selective adsorption of Ga^3+^ from aqueous solutions.

The adsorption selectivity of the GO-AHZTA composite toward Ga^3+^, Sc^3+^ and In^3+^ was evaluated. In comparison, *q_e_* values of 23.92, 4.87 and 1.90 mg g^−1^ for Ga^3+^, Sc^3+^ and In^3+^, respectively, on the GO-AHZTA composite were obtained suggesting that the developed adsorbent possesses higher adsorption selectivity toward Ga^3+^ than toward the other two metal ions (Figure 6B). Previous research also indicated that triazole possessed a higher affinity toward Ga^3+^ than toward Sc^3+^ and In^3+^ due to metal–electron donor interactions [42]. In the following batch adsorption experiments, Ga^3+^ was utilized as the model adsorbate to study the effect of operating conditions such as contact time, initial solution pH, initial Ga^3+^ concentration and contact temperature on the adsorption process.

#### 2.2.2. Effect of Solution pH

**Figure 7:**
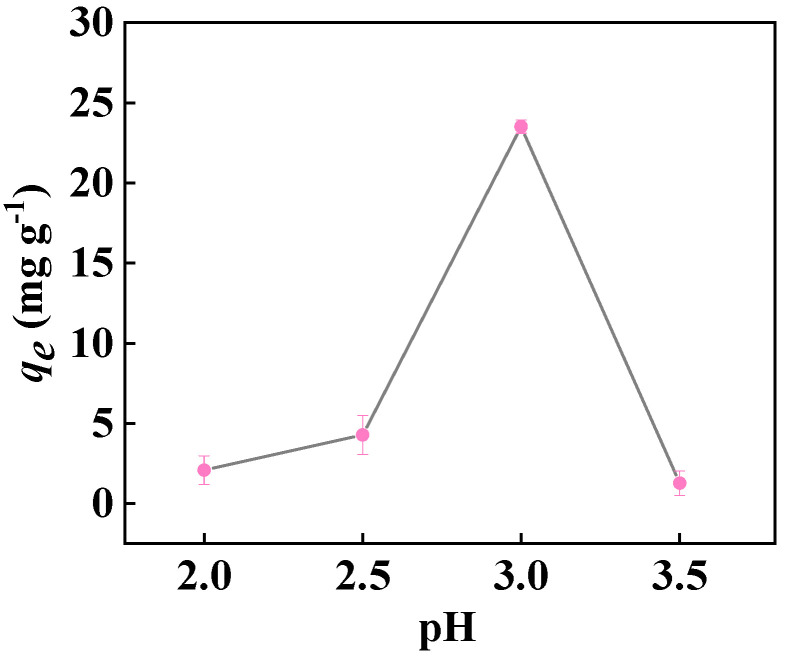
Effects of solution pH on the adsorption of Ga^3+^ by the GO-AHZTA composite (*C*_0_ = 50.0 mg L^−1^; pH = 3.0; T = 25 °C; adsorbent dosage = 5.0 mg).

The solution pH is an important parameter that controls the adsorption process. As reported previously, Ga^3+^ precipitates at pH ~3.5 and redissolves at pH ~8 [43]; that is, Ga^3+^ hydroxide precipitates in the pH range ~3.7–7.0. Therefore, adsorption experiments were carried out in the pH range 2–3.5 (Figure 7). The amount of Ga^3+^ adsorbed by the GO-AHZTA composite increased sharply from 2.9 to 23.916 mg g^−1^ as the pH increased from 2.0 to 3.0, then quickly decreased to 1.29 mg g^−1^ at pH = 3.5. The adsorption capacity of the GO-AHZTA composite is very small at lower pH values mainly due to the competition of H_3_O^+^ with Ga^3+^ for active sites. At the higher pH value of 3.5, the formation of [Ga(OH)]^2+^ and [Ga(OH)_2_]^+^ will weaken the interactions with GO-AHZTA composite, causing a decrease in the adsorption capacity [44].

#### 2.2.3. Effect of Time and Adsorption Kinetics

**Figure 8:**
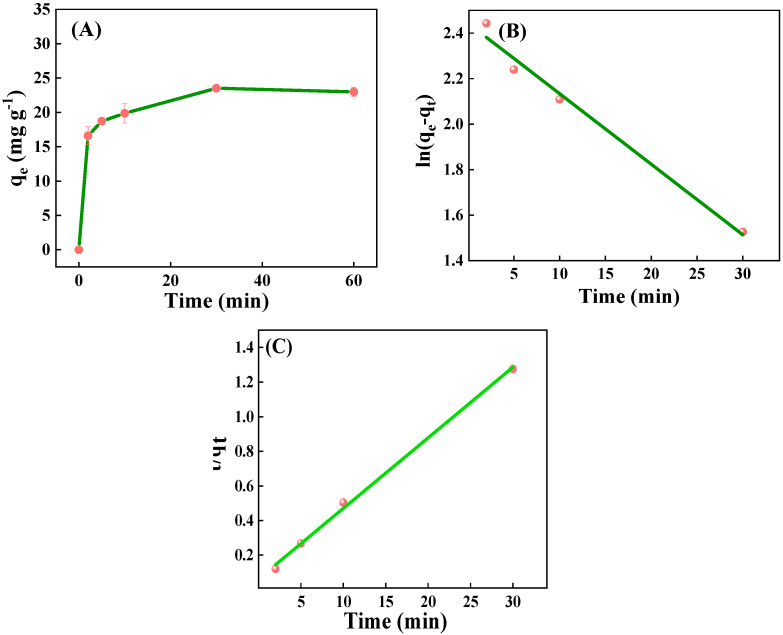
The adsorption properties of the GO-AHZTA composite toward Ga^3+^. (**A**) Effect of contact time (*C_0_* = 50.0 mg L^−1^; pH = 3.0; T = 25 °C). (**B**) Experimental data fitted to the linear pseudo-first-order kinetic model; (**C**) Experimental data fitted to the linear pseudo-second-order kinetic model.

As an important factor that defines the effectiveness of an adsorption process, kinetics can basically describe the rate and the retention time of the adsorbates on the solid–liquid interface [45]. The influence of the adsorption time on the GO-AHZTA composite toward Ga^3+^ was studied; the adsorption equilibrium could be achieved in 30 min (Figure 8A). The efficient adsorption could be attributed to the availability of active sites on the surface that directly contributes to the adsorbent–adsorbate interactions [46]. Various kinetic models, including linear pseudo-first-order and linear pseudo-second-order models, were adopted to fit the experimental data (Equations (S1) and (S2)). As shown in Figure 8B,C and Table 1, the adsorption data fit best with the linear pseudo-second order kinetic model, as shown by the higher coefficient of determination (*R*^2^ = 0.997). In addition, the calculated *q_e_* value (24.534 mg g^−1^) is very close to the experimental result (23.916 mg g^−1^).

#### 2.2.4. Effects of Initial Concentration and Contact Temperature, and Adsorption Isotherms

**Figure 9:**
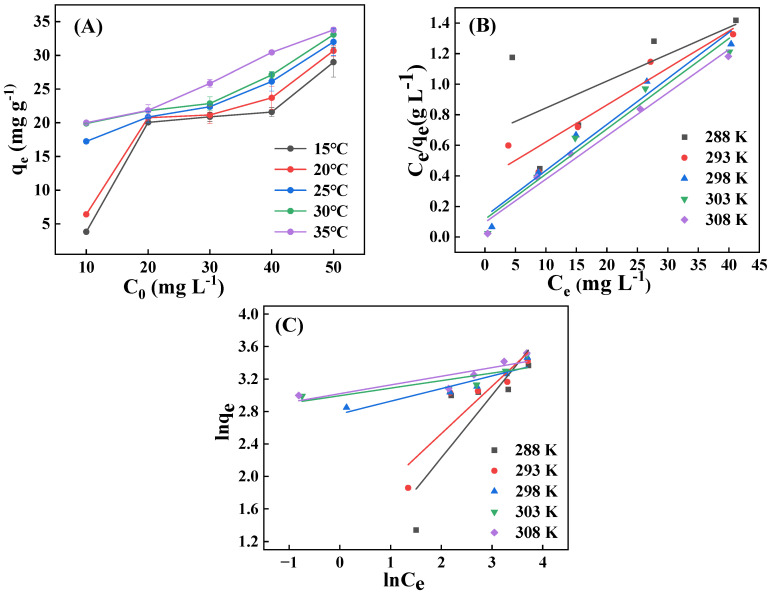
The adsorption properties of the GO-AHZTA composite toward Ga^3+^. (**A**) Effect of initial concentration and temperature on the adsorption capacity of the GO-AHZTA composite. (**B**) Experimental data are fitted to the linear Langmuir isotherm. (**C**) Experimental data fitted to the linear Freundlich isotherm.

The equilibrium uptake amount (mg g^−1^) versus the initial Ga^3+^ concentration (10, 20, 30, 40 and 50 mg L^−1^) at various contact temperatures in the range 288–303 K were recorded (Figure 9A). The amount (mg g^−1^) of Ga^3+^ adsorbed increased with an increase in the initial concentration at all the tested temperatures, indicating that the adsorption of Ga^3+^ depends on its initial concentration, which might provide one of the most important driving forces to overcome the liquid-phase mass-transfer resistance [47]. In addition, the equilibrium uptake amount (mg g^−1^) of the GO-AHZTA composite for Ga^3+^ increases with an increase in the contact temperature, suggesting that an endothermic reaction during the adsorption process occurs. Equilibrium relationships between the GO-AHZTA composite and Ga^3+^ are described by different adsorption isotherms, including the linear Langmuir isothermal and linear Freundlich isothermal models (Equations (S3) and (S4)) [48]. As shown in Figure 9B,C and Table 2, the parameters of different isothermal models are summarized. Clearly, the linear Langmuir isothermal model can adequately represent the experimental adsorption data, based on the values of the coefficient of determination (*R*^2^), suggesting that monolayer adsorption occurs because the active sites are present on the surface [49]. The values of the parameter, 0 < *k_L_* < 1, indicate that the adsorption system is favorable [50].

#### 2.2.5. Adsorption Thermodynamics

Furthermore, the thermodynamic parameters of the GO-AHZTA composite for Ga^3+^, such as the change in the enthalpy (Δ*H*°, kJ·mol^−1^), the change in the entropy (Δ*S^o^*, J K^−1^ mol^−1^) and the change in the Gibbs free energy (Δ*G*°, kJ mol^−1^) were calculated (Equations (S5)–(S7), Figure S2, Table S1). The Gibbs free energy change reflects the system’s stability in adsorption thermodynamics. At initial Ga^3+^ concentrations < 50 mg L^−1^, Δ*G*° values are almost all negative, indicating that the adsorption is spontaneous, which is consistent with the results obtained by the adsorption isotherms. The positive Δ*H^o^* values suggest that the adsorption is endothermic, and increasing the contact temperature is beneficial for improving the adsorption efficiency. Additionally, the relatively large value (>40 kJ mol) at the initial Ga^3+^ concentration of 10 mg L^−1^ suggests that chemical adsorption dominates the adsorption of Ga^3+^ by the GO-AHZTA composite [51]. The positive value of Δ*S*° confirms the affinity of the GO-AHZTA composite for Ga^3+^ because an increase in randomness at the solid/solution interface was generated, indicating that Ga^3+^ tends to rapidly aggregate on the surface of the GO-AHZTA composite in a random manner due to its roughness and the unevenly distributed binding sites [52], which is consistent with the results discussed previously.

#### 2.2.6. Proposed Adsorption Mechanism

**Figure 10:**
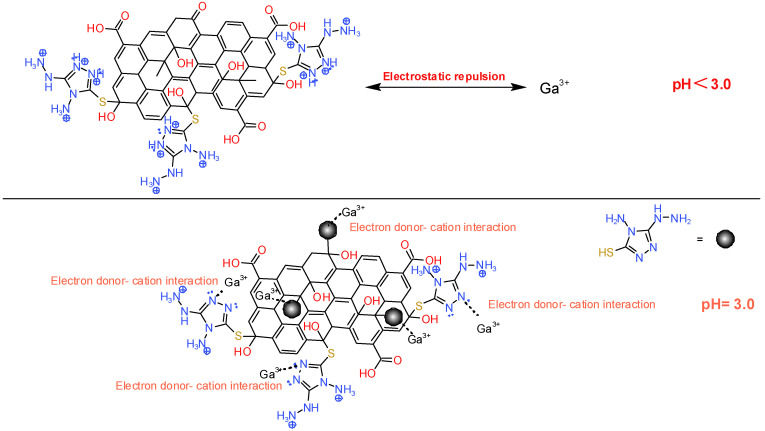
The proposed adsorption mechanism of the GO-AHZTA composite toward Ga^3+^.

Metal ions can selectively interact with specific ligands to form coordination compounds. Overall, the GO-AHZTA composite possesses aromatic cycles and oxygen (O)-/nitrogen (N)-/sulfur (S)-containing groups that might form electron donor and acceptor (cation) interactions. Nonetheless, N-containing groups, especially the N-N bond of 1,2,4-thiazole, are more likely to dominate the adsorption capacity for Ga^3+^ (Figure 10) [18,22]. The amino, N-N, and hydrazine groups would be intensely protonated under low pH (pH < 3), producing electrostatic repulsions between Ga^3+^ and the GO-AHZTA composite, resulting in relatively lower adsorption capacity. At relatively higher pH (>3), the formation of [Ga(OH)]^2+^ and [Ga(OH)_2_]^+^ will also result in decreased adsorption capacity due to the weakened electrostatic attractions. As previously mentioned, the unprotonated N-N bond could selectively interact with Ga^3+^ through electron donor–cation interactions at a suitable solution pH of 3.0. Therefore, monolayer adsorption due to the complexation of the N-N group of the GO-AHZTA composite and Ga^3+^ plays the most crucial role in the adsorption. 

### 2.3. Desorption and Regeneration

The regeneration of the GO-AHZTA composite and the elution of Ga^3+^ from the adsorbent are crucial indicators in practical applications. The desorption experiments were carried out by using aqueous HCl and NaOH solutions as the eluents. Interestingly, either 1.0 mol·L^−1^ HCl or 1.0 mol·L^−1^ NaOH could be used for the desorption/regeneration processes. The GO-AHZTA composite maintained good adsorption performance (>80%) after five experimental cycles using 1.0 mol·L^−1^ NaOH as the eluent, while it maintained a relatively lower adsorption performance (>60%) after five experimental cycles using 1.0 mol·L^−1^ HCl as the eluent, indicating that the GO-AHZTA composite is more stable under alkaline conditions. Therefore, the GO-AHZTA composite can be used as an efficient adsorbent for the aqueous adsorption of Ga^3+^.

### 2.4. Adsorption Performance in a Mixed Solution

The adsorption performance of the GO-AHZTA composite (5.0 mg) for a mixed solution (20.00 mL) containing Ga^3+^ (20.0 mg L^−1^), Sc^3+^ (20.0 mg L^−1^) and In^3+^ (20.0 mg L^−1^) at 25 °C was investigated. After oscillating the mixture in a conical flask (100.0 mL) for 180 min, the concentrations of Ga^3+^, Sc^3+^ and In^3+^ were determined by an ICP-OES. The relatively lower adsorption capacity of 4.84, 1.89 and 0 mg mL^−1^ for Ga^3+^, Sc^3+^ and In^3+^, respectively, was obtained. Obviously, the GO-AHZTA composite possesses high adsorption selectivity toward Ga^3+^ even in the complex solution. The adsorption capacity of the GO-AHZTA composite in the mixed solution is significantly lower than that (20.84 mg L^−1^) in the solution containing only Ga^3+^, indicating that the coexistence of other metal ions will greatly impede the migration of ions, thus increasing the activation energy of ion migration and decreasing the adsorbability of the adsorbent [53].

## 3. Experimental Details

### 3.1. Reagents and Materials

Flake graphite (80–90.5 wt.%) was provided by Qingdao Braide Graphite Co., Ltd. (Qingdao, China). Potassium permanganate (KMnO_4_) and phosphoric acid (H_3_PO_4_, 85 wt.%) were supplied by Sinopharm Group Chemical Reagent Co., Ltd. (Shanghai, China). Hydrogen peroxide (H_2_O_2_, 30.0 wt.%) was purchased from Shanghai Wokai Biotechnology Co., Ltd. (Shanghai, China). Concentrated sulfuric acid (H_2_SO_4_, 98 wt.%), hydrochloric acid (HCl, 37 wt.%) and sodium hydroxide (NaOH; AR, 97 wt.%) were provided by Chengdu Cologne Chemical Co., Ltd. (Chengdu, China); 4-amino-3-hydrazino-1,2,4-triazol-5-thiol (AHTZT, C_2_H_6_N_6_S, 99 wt.%) was bought from Shanghai Aladdin Bio-Chem Technology Co., Ltd. (Shanghai, China); scandium nitrate hydrate (Sc(NO_3_)_3_∙H_2_O; 99.9 wt.%), indium chloride (InCl_3_, 99.99%) and gallium nitrate hydrate (Ga(NO_3_)_3_∙H_2_O; 99.99 wt.%) were purchased from Shanghai Run-Biotech Co., Ltd. (Shanghai, China). Ultrapure water with a resistivity of 18.2 MΩ cm^−1^ was produced from a Millipore Milli-Q water purification system (Millipore Trading Co., Ltd.; Shanghai, China). All chemicals were of analytical grade and used without further purification. 

### 3.2. Preparation of the GO-AHTZT Composite

The facile preparation of GO powders was carried out by referring to the modified Hummer’s method [54,55]. Generally, flake graphite (0.60 g) was blended with KMnO_4_ (3.00 g) to obtain a homogeneous mixture, which was transferred into a 500.0 mL round-bottom bottle and then immersed in an ice-water bath. A mixed acid of H_3_PO_4_ (85%, 8.0 mL) and H_2_SO_4_ (98%, 72.0 mL) was added dropwise to the reaction under vigorous and constant stirring. After the dispersion gradually turned green, it was heated to 50 °C for another 12 h until it turned purple-red. The reaction was naturally cooled to room temperature, and excess H_2_O_2_ was added. The dispersion finally turned bright yellow, and bubbles were no longer produced. The dispersion was rinsed repeatedly with ultrapure water/HCl (1.0 mol L^−1^) and centrifuged until the pH value of the supernatant was neutral. The residual black solid was collected and dispersed in 20.0 mL of ultrapure water and freeze-dried at −50 °C for 96 h to produce the GO powders.

GO (500.0 mg), ultrapure water (50.0 mL) and concentrated H_2_SO_4_ (98 wt.%; 1.0 mL) were placed in a round-bottom flask (100.0 mL), then ultrasonically treated to produce a well-dispersed mixture. After 1.00 g of AHZTA was added into the reaction, it was heated to 110 °C for 8 h in an oil bath, then naturally cooled to room temperature. The mixture was diluted with a certain amount of ultrapure water, then filtered and washed repeatedly with ultra-pure water and ethanol for at least three times until the filtrate was close to neutral. The black solid on the filter paper was carefully collected and dispersed in a certain amount (20.0 mL) of ultrapure water, then freeze-dried for 72 h to obtain the GO-AHZTA composite.

### 3.3. Characterization of the GO-AHZTA Composite

To detect the surface functional groups of the AHZTA and GO-AHZTA composites, Fourier transform–infrared (FT-IR) spectra were recorded on a Shimadzu FT-IR spectrophotometer (IR Prestige-21; Shimadzu, Ltd., Tokyo, Japan) in the wavenumber range 4000–400 cm^−1^ with a resolution of 4 cm^−1^. The morphology and elemental distribution of the GO-AHZTA composite pre- and post-Ga^3+^ adsorption were investigated by a scanning electron microscope (SEM; JSM-7900F, JEOL Corp.; Tokyo, Japan) equipped for energy dispersive X-ray spectroscopy (EDS) at an accelerating voltage of 10 kV. To determine the compositions, chemical states and possible metal–chelate bonding interactions of the GO-AHZTA composite post-Ga^3+^ adsorption, X-ray photoelectron spectroscopy (XPS) was performed on a Perkin Elmer PHI 5000 C ESCA instrument (Perkin Elmer Co.; Eden Prarie, MN, USA) using Al Kα radiation (1486.6 eV) with a detection angle of 54° and a cathode voltage of 14 kV, which is operated at 250 W. The concentration of metal ions in the solutions pre- and post-adsorption was determined on an inductively coupled plasma–optical emission spectrometer (ICP-OES; ICAP PRO X, Thermo Fisher Scientific Inc.; Walthan, MA, USA). Thermogravimetric analysis (TGA) measurements for the samples in powder form were conducted using a STA449 F3 thermogravimetric analyzer (Netzsch, Selb, Germany) at a heating rate of 10 °C/min under an argon (Ar) atmosphere and a flow rate of 50 mL/min.

### 3.4. Adsorption Experiments

Several metal ion solutions that are present in the leaching of VSPR, namely Ga^3+^, Sc^3+^ and In^3+^, were selected to evaluate the adsorption performances of the GO-AHZTA composite and GO. Oscillation of the adsorbent (GO-AHZTA composite or GO, 5.0 mg) in metal ion solution (20.0 mL, 50.0 mg L^−1^) in a conical bottle (100.0 mL) was implemented for 180 min at 25 °C. Three parallel experiments were performed, and the adsorption equilibrium adsorption capacity (*q_e_*; mg g^−1^) was calculated according to Equation (1):

(1) $$q_{e}=\frac{(C_{0}-C_{e})\cdot V}{m}$$

where *C*_0_ (mg L^−1^) and *C_e_* (mg L^−1^) are the initial Ga^3+^ concentration and the Ga^3+^ concentration after adsorption; *V* (L) is the volume of the Ga^3+^ solution; *m* (g) is the mass of the adsorbent and *q_e_* (mg g^−1^) represents the equilibrium adsorption capacity of the adsorbent.

### 3.5. Batch Adsorption Experiments

An appropriate amount of Ga(NO_3_)_3_∙H_2_O was dissolved in ultrapure water, transferred to a volumetric flask and diluted to the desired volume to produce the Ga^3+^ solutions. The effects of contact time, initial Ga^3+^ concentration, contact temperature, as well as initial solution pH value on the adsorption properties of the GO-AHZTA composite toward Ga^3+^ were studied in detail. The continuous oscillation of the GO-AHZTA composite (5.0 mg) in Ga^3+^ solution (20.0 mL) was carried out in a 100.0 mL conical flask at a certain temperature (15 °C, 20 °C, 25 °C, 30 °C, and 35 °C) for specified times (0–140 min). Samples were taken out every 20 min, and the Ga^3+^ concentration was measured using an ICP-OES (PerkinElmer Optima 4300DV; PerkinElmer Inc., Boston, MA, USA).

## 4. Conclusions

A novel GO-based adsorbent, the GO-AHTZT composite, was developed by a nucleophilic addition reaction. The samples were characterized by various techniques such as SEM, EDS, elemental mapping, XPS, TGA and FT-IR spectroscopy. The adsorption capacity (23.92 mg g−1) of the GO-AHZTA composite toward Ga3+ is 4.9 and 12.6 times greater than those toward Sc3+ and In3+, respectively, which facilitates the selective adsorption of Ga3+ from aqueous solutions. Adsorption conditions, including contact time, solution pH, initial Ga3+ concentration and contact temperature, were investigated. In addition, a possible adsorption mechanism was also proposed. The adsorption data was fitted well by the linear pseudo-second order kinetic model and linear Langmuir isothermal model, suggesting that monolayer adsorption occurs on the surface. The thermodynamic analysis confirms that it is an endothermic chemical adsorption process.

## Figures and Tables

**Table 1:** Kinetic parameters for the adsorption of Ga^3+^ onto the GO-AHZTA composite.

Adsorption Kinetic Model	*R* ^2^	*k*_1_ (min^−1^)	*k*_2_ (min^−1^)	*q_e_* (mg g^−1^)
Linear pseudo-first-order	0.985	0.031	-	6.641
Linear pseudo-second order	0.997	-	0.0264	24.534

**Table 2:** Adsorption isothermal parameters for the adsorption of Ga^3+^ onto the GO-AHZTA composite.

*T* (K)	Linear Langmuir Model			Linear Freundlich Model		
	*R* ^2^	*k_L_*	*q_m_* (mg g^−1^)	*R* ^2^	*k_F_*	*n*
288	0.416	0.026	56.850	0.708	1.869	1.295
293	0.898	0.064	41.356	0.809	3.684	1.709
298	0.964	0.225	33.211	0.863	7.530	6.411
303	0.961	0.256	33.818	0.650	8.141	10.778
308	0.980	0.304	34.722	0.752	8.208	9.320

## Data Availability

Data are contained within the article and supplementary materials.

## Supplementary Materials

The following supporting information can be downloaded at: https://www.mdpi.com/article/10.3390/molecules29122778/s1, Figure S1: FT-IR spectra of GO; Figure S2: Experimental data and the fitted curve of Ink_d_ versus 1/T calculated from Van’t Hoff plots of GO-AHZTA composite for Ga^3+^ with different concentration: (A) 10 mg L^−1^; (B) 20 mg L^−1^; (C) 30 mg L^−1^; (D) 40 mg L^−1^; (E) 50 mg L^−1^; Table S1: Thermodynamic parameters of the adsorption by GO-AHZTA composite.

## References

[1] Syu C.-H., Chen L.-Y., Lee D.-Y. (2021). The growth and uptake of gallium (Ga) and indium (In) of wheat seedlings in Ga- and In-contaminated soils. Sci. Total Environ..

[2] Wood S.A., Samson I.M. (2006). The aqueous geochemistry of gallium, germanium, indium and scandium. Ore Geol. Rev..

[3] Ergashev S. (2023). Anomalously high diotovoltaic effect in thin films of gallium arsenide. Int. J. Adv. Sci. Res..

[4] Tarbi A., Chtouki T., Bouich A., Elkouari Y., Erguig H., Migalska-Zalas A., Aissat A. (2022). InP/InGaAsP thin films based solar cells: Lattice mismatch impact on efficiency. Opt. Mater..

[5] Moskalyk R.R. (2003). Gallium: The backbone of the electronics industry. Miner. Eng..

[6] Zheng K., Benedetti M.F., van Hullebusch E.D. (2023). Recovery technologies for indium, gallium, and germanium from end-of-life products (electronic waste)–A review. J. Environ. Manag..

[7] Du J., Zhang M., Dong Z., Yang X., Zhao L. (2022). Facile fabrication of tannic acid functionalized microcrystalline cellulose for selective recovery of Ga (III) and In (III) from potential leaching solution. Sep. Purif. Technol..

[8] Hu D., Ma B., Li X., Lv Y., Zhang W., Chen Y., Wang C. (2022). Efficient separation and recovery of gallium and indium in spent CIGS materials. Sep. Purif. Technol..

[9] Fabretti A.C., Franchini G.C., Peyronel G. (1982). Cobalt(II), nickel(II), copper(II) and copper(I) complexes of 2-mercapto-5-methyl-1,3,4-thiadiazole and 2,5-bis(methylmercapto)-1,3,4-thiadiazole. Transit. Met. Chem..

[10] Bharati P., Bharti A., Bharty M.K., Kashyap S., Singh U.P., Singh N.K. (2013). Synthesis, spectral and structural characterization of Ni(II), Cu(II), Zn(II), Cd(II) and Hg(II) complexes with 2-mercapto-5-methyl-1,3,4-thiadiazole: A Zn(II) complex acting as a new sensitive and selective fluorescent probe for the detection of Hg2+ in H2O–MeOH medium. Polyhedron.

[11] Raj P., Patel M., Karamalidis A.K. (2023). Chemically modified polymeric resins with catechol derivatives for adsorption, separation and recovery of gallium from acidic solutions. J. Environ. Chem. Eng..

[12] Cui J., Cong X., Li X., Chen X., Wang Y., Gao J., Xiong Y. (2023). Chitosan derived layered porous carbon and its performance on gallium adsorption. J. Chem. Technol. Biotechnol..

[13] Li S., Fan J., Gao L. (2023). Conductive biomass carbon aerogel with high adsorption performance for gallium in alkaline solution. Miner. Eng..

[14] Jankovský O., Šimek P., Klímová K., Sedmidubský D., Pumera M., Sofer Z. (2015). Highly selective removal of Ga^3+^ ions from Al^3+^/Ga^3+^ mixtures using graphite oxide. Carbon.

[15] Deshwal N., Singh M.B., Bahadur I., Kaushik N., Kaushik N.K., Singh P., Kumari K. (2023). A review on recent advancements on removal of harmful metal/metal ions using graphene oxide: Experimental and theoretical approaches. Sci. Total Environ..

[16] Li X., Zhang J., Pang Z., Zhu Y., Chen X., Sun Q., Li Y. (2019). Photoelectrocatalytic decolorization of methylene blue using reduced graphene oxide modified TiO2 on filter paper. Water Sci. Technol..

[17] Zhang Y., Liu X., Wang Y., Lou Z., Shan W., Xiong Y. (2019). Polyacrylic acid-functionalized graphene oxide for high-performance adsorption of gallium from aqueous solution. J. Colloid Interface Sci..

[18] Ji Q., Hu C., Liu H., Qu J. (2018). Development of nitrogen-doped carbon for selective metal ion capture. Chem. Eng. J..

[19] Xu W., Zhou S., Wang B., Zhang P., Tang K. (2022). Efficient adsorption of Au (III) from acidic solution by a novel N, S-containing metal–organic framework. Sep. Purif. Technol..

[20] Deng J., Liu Y., Liu S., Zeng G., Tan X., Huang B., Tang X., Wang S., Hua Q., Yan Z. (2017). Competitive adsorption of Pb (II), Cd (II) and Cu (II) onto chitosan-pyromellitic dianhydride modified biochar. J. Colloid Interface Sci..

[21] Yin Z., Hu Y., Sun W., Zhang C., He J., Xu Z., Zou J., Guan C., Zhang C., Guan Q. (2018). Adsorption mechanism of 4-amino-5-mercapto-1, 2, 4-triazole as flotation reagent on chalcopyrite. Langmuir.

[22] Wang L., Cheng J., Liu N., Zou H., Yan H., Lu J., Liu H., Li Y., Dou J., Wang S. (2023). Two Co-based metal–organic framework isomers with similar metal-carboxylate sheets: Turn-on ratiometric luminescence sensing activities toward biomarker N-acetylneuraminic acid and discrimination of Ga^3+^ and In^3+^. Inorg. Chem..

[23] Brahma G.S., Mohanty P. (2003). Kinetics and mechanism of complex formation between pentamminesuccinatocobalt (III) and gallium (III). Pol. J. Chem..

[24] Mateo C., Grazu V., Palomo J.M., Lopez-Gallego F., Fernandez-Lafuente R., Guisan J.M. (2007). Immobilization of enzymes on heterofunctional epoxy supports. Nat. Protoc..

[25] Grazú V., Abian O., Mateo C., Batista-Viera F., Fernández-Lafuente R., Guisán J.M. (2003). Novel bifunctional epoxy/thiol-reactive support to immobilize thiol containing proteins by the epoxy chemistry. Biomacromolecules.

[26] Cote L.J., Kim J., Zhang Z., Sun C., Huang J. (2010). Tunable assembly of graphene oxide surfactant sheets: Wrinkles, overlaps and impacts on thin film properties. Soft Matter.

[27] Ru J., Wang X., Wang F., Cui X., Du X., Lu X. (2021). UiO series of metal-organic frameworks composites as advanced sorbents for the removal of heavy metal ions: Synthesis, applications and adsorption mechanism. Ecotoxicol. Environ. Saf..

[28] Jiang H., Yang Y., Lin Z., Zhao B., Wang J., Xie J., Zhang A. (2020). Preparation of a novel bio-adsorbent of sodium alginate grafted polyacrylamide/graphene oxide hydrogel for the adsorption of heavy metal ion. Sci. Total Environ..

[29] Branca C., D’Angelo G., Crupi C., Khouzami K., Rifici S., Ruello G., Wanderlingh U. (2016). Role of the OH and NH vibrational groups in polysaccharide-nanocomposite interactions: A FTIR-ATR study on chitosan and chitosan/clay films. Polymer.

[30] Silva A.L.R., Gonçalves J.M., Morais V.M.F., Ribeiro da Silva M.D.M.C. (2022). Thermodynamic properties of 2-mercapto-, 2,5-dimethyl- and 2-mercapto-5-methyl-1,3,4-thiadiazole. J. Chem. Thermodyn..

[31] Ren F., Li Z., Tan W.Z., Liu X.H., Sun Z.F., Ren P.G., Yan D.X. (2018). Facile preparation of 3D regenerated cellulose/graphene oxide composite aerogel with high-efficiency adsorption towards methylene blue. J. Colloid Interf. Sci..

[32] Miyata O., Shinada T., Ninomiya I., Naito T., Date T., Okamura K., Inagaki S. (1991). Stereospecific nucleophilic addition reactions to olefins. Addition of thiols to. a,b-unsaturated carboxylic acid derivatives. J. Org. Chem..

[33] Ramana C.V., Rubio E.J., Barraza C.D., Miranda Gallardo A., McPeak S., Kotru S., Grant J.T. (2014). Chemical bonding, optical constants, and electrical resistivity of sputter-deposited gallium oxide thin films. J. Appl. Phys..

[34] Zhang J., Chen L., Yang J., Bian C., He W. (2023). Thermal behaviors, thermal decomposition mechanism, kinetic model analysis and thermal hazard prediction of 3,6,7-triamino-7H-[1,2,4]triazolo [4,3-b][1,2,4]triazole (TATOT). Thermochim. Acta.

[35] Chen X., Wang X., Fang D. (2020). A review on C1s XPS-spectra for some kinds of carbon materials. Fuller. Nanotub. Carbon Nanostruct..

[36] Briggs D., Beamson G. (1993). XPS studies of the oxygen 1s and 2s levels in a wide range of functional polymers. Anal. Chem..

[37] Poolwong J., Del Gobbo S., D’Elia V. (2021). Transesterification of dimethyl carbonate with glycerol by perovskite-based mixed metal oxide nanoparticles for the atom-efficient production of glycerol carbonate. J. Ind. Eng. Chem..

[38] Tangyen N., Natongchai W., Del Gobbo S., D’Elia V. (2024). Revisiting the potential of group Vi inorganic precatalysts for the ethenolysis of fatty acids through a mechanochemical approach. ACS Omega.

[39] Wang R., Huang J., Deng Y., Fan K., Xu B., Zhao Z. (2024). Preparation and properties of protein-derived carbon-modified CoS2 applied to high-performance lithium-sulfur battery. Mater. Des..

[40] Farivar F., Yap P.L., Hassan K., Tung T.T., Tran D.N., Pollard A.J., Losic D. (2021). Unlocking thermogravimetric analysis (TGA) in the fight against “Fake graphene” materials. Carbon.

[41] Li W., Zhou C., Li C., Zhu W., Shi J., Liu G. (2023). Synthesis of UiO-66 series metal–organic framework composites and the adsorption effect on gallium. Chem. Eng. J..

[42] Alhamami M.A.M., Algethami J.S., Khan S. (2023). A review on thiazole based colorimetric and fluorimetric chemosensors for the detection of heavy metal ions. Crit. Rev. Anal. Chem..

[43] Kubicek V., Havlickova J., Kotek J., Tircsó G., Hermann P., Tóth É., Lukes I. (2010). Gallium (III) complexes of DOTA and DOTA−monoamide: Kinetic and thermodynamic studies. Inorg. Chem..

[44] Simecek J., Schulz M., Notni J., Plutnar J., Kubicek V., Havlickova J., Hermann P. (2012). Complexation of metal ions with TRAP (1, 4, 7-triazacyclononane phosphinic acid) ligands and 1,4,7-triazacyclononane-1,4,7-triacetic acid: Phosphinate-containing ligands as unique chelators for trivalent gallium. Inorg. Chem..

[45] Wang J., Guo X. (2020). Adsorption kinetic models: Physical meanings, applications, and solving methods. J. Hazard. Mater..

[46] Nithya K., Sathish A., Senthil Kumar P., Ramachandran T. (2018). Fast kinetics and high adsorption capacity of green extract capped superparamagnetic iron oxide nanoparticles for the adsorption of Ni(II) ions. J. Ind. Eng. Chem..

[47] Mozaffari Majd M., Kordzadeh-Kermani V., Ghalandari V., Askari A., Sillanpää M. (2022). Adsorption isotherm models: A comprehensive and systematic review (2010−2020). Sci. Total Environ..

[48] Yi Q., Hao X., Li X., Dong H., Sun L. (2023). Effect of TiO_2_ nanoparticles on the mass transfer process of absorption of toluene: Experimental investigation and molecular dynamics simulation. J. Environ. Chem. Eng..

[49] Mujtaba G., Ullah A., Khattak D., Shah M.U.H., Daud M., Ahmad S., Hai A., Ahmed F., Alshahrani T., Banat F. (2023). Simultaneous adsorption of methylene blue and amoxicillin by starch-impregnated MgAl layered double hydroxide: Parametric optimization, isothermal studies and thermo-kinetic analysis. Environ. Res..

[50] Chen H., Zhao J., Wu J., Dai G. (2011). Isotherm, thermodynamic, kinetics and adsorption mechanism studies of methyl orange by surfactant modified silkworm exuviae. J. Hazard. Mater..

[51] Fang Y., Liu Q., Song Y., Jia F., Yang Y., Li H. (2023). Rational design of anti-interference Fe/Co MOF-coupled PMS process for As (III) removal in DOM-rich groundwater: 1O2-dominated As (III) oxidation and chemisorption of As (V). Chem. Eng. J..

[52] Hong S., Wen C., He J., Gan F.X., Ho Y.S. (2009). Adsorption thermodynamics of Methylene Blue onto bentonite. J. Hazard. Mater..

[53] Olsson E., Chai G., Dove M., Cai Q. (2019). Adsorption and migration of alkali metals (Li, Na, and K) on pristine and defective graphene surfaces. Nanoscale.

[54] Chen D., Feng H., Li J. (2012). Graphene oxide: Preparation, functionalization, and electrochemical applications. Chem. Rev..

[55] Shen J., Hu Y., Shi M., Lu X., Qin C., Li C., Ye M. (2009). Fast and facile preparation of graphene oxide and reduced graphene oxide nanoplatelets. Chem. Mater..

