# Supplementary material for: Preparation of 4-Amino-3-hydrazino-1,2,4-triazol-5-thiol-Modified Graphene Oxide and Its Greatly Enhanced Selective Adsorption of Gallium in Aqueous Solution
Source: Molecules. 2024 Jun 11;29(12):2778. doi: 10.3390/molecules29122778 (PMC11206100; doi:10.3390/molecules29122778)
Supplement: Supplementary file 1 [file molecules-29-02778-s001.zip › molecules-3018675-supplementary.pdf]

Supporting Information

Preparation of 4-amino-3-hydrazino-1,2,4-triazol-5-thiol-modified graphene oxide and its greatly enhanced selective adsorption of gallium in aqueous solution

Xi Zhu, Yong Guo* Baozhan Zheng

College of Chemistry，Sichuan University, Chengdu 610065, China

1. FT-IR spectra of GO

As shown in **Fig. S1**, the peaks at 3391.8 and 1721.2 cm^{-1} can be attributed to the stretching vibrations of the hydroxyl (–OH) and C=O groups of GO, respectively. In addition, the peak at 1622.1 cm^{-1} can be assigned to the C=O stretching vibrations of carboxyl groups and the peak at 1567.9 corresponds to aromatic skeletal vibrations, while the peaks at 1229.8 and 1037.5 cm^{-1} belong C–O and C-C stretching vibrations, respectively [1].

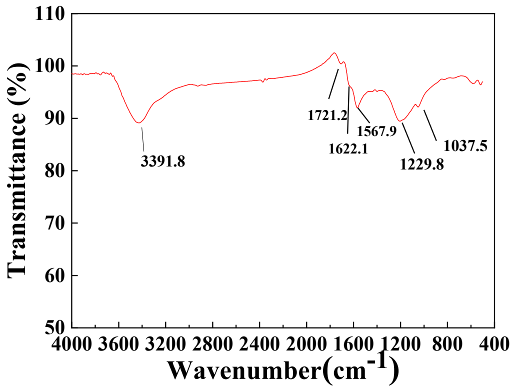

Fig. S1 FT-IR spectra of GO.

2. Adsorption kinetic models

To whom correspondence should be addressed.
* Y Guo, E-mail: guoy@scu.edu.cn;

The two kinetic models can expressed as follows (**Eq. S1-S2**):

Linear pseudo-first-order model: $\ln(q_e - q_t) = \ln q_e - k_1 t$ (S1)

Linear pseudo-second-order model: $\frac{t}{q_t} = \frac{1}{k_2 q_e^2} + \frac{t}{q_e}$ (S2)

where q_e (mg g^{-1}) and q_t (mg g^{-1}) are the amounts of Ga^{3+} adsorbed by the GO-AHZTA composite in the equilibrium state and at contact time *t* (min), respectively, and k_1 (min^{-1}) and k_2 (g mg^{-1} min^{-1}) are the specific adsorption rate constant of the pseudo-first-order model and the pseudo-second-order model, respectively.

3. Adsorption isothermal models

The isothermal models are represented by the following equations (**Eq. S3-S4**):

Linear Langmuir isothermal model: $\frac{C_e}{q_e} = \frac{C_e}{q_m} + \frac{1}{q_m k_L}$ (S3)

Linear Freundlich isothermal model: $\ln q_e = \ln k_F + \frac{1}{n} \ln C_e$ (S4)

where C_e (mg L^{-1}) is the equilibrium concentration of Ga^{3+}; q_e (mg g^{-1}) is the amount of Ga^{3+} adsorbed by the GO-AHZTA composite in the equilibrium state; q_m (mg g^{-1}) is the monolayer adsorption capacity and k_L (L mg^{-1}) is the Langmuir equilibrium constant. Of the two Freundlich constants, *n* represents the relative advantage of adsorption process, while k_F (mg L$^{1/n}$ g^{-1} mg$^{-1/n}$) is defined as the adsorption or distribution coefficient that corresponds to the amount of Ga^{3+} adsorbed onto the GO-AHZTA composite at the unit equilibrium concentration. The slope 1/n, ranging between 0 and 1, can used as a measure of adsorption intensity or surface heterogeneity, with a value close to zero suggesting a more heterogeneous surface [2].

4. Adsorption thermodynamics

The change in the enthalpy (ΔH^o, kJ mol^{-1}), the change in the entropy (ΔS^o, J K^{-1} mol^{-1}) and the change in the Gibbs free energy (ΔG^o, kJ mol^{-1}) can be calculated by **Eq. S5-S7**.

$$k_d = \frac{C_0 - C_e}{C_e} \times \frac{V}{m} \qquad \text{(S5)}$$

$$\ln k_d = \frac{-\Delta G^o}{RT} = \frac{\Delta S^o}{R} - \frac{\Delta H^o}{RT} \qquad \text{(S6)}$$

$$\Delta G^o = \Delta H^o - T\Delta S^o \qquad \text{(S7)}$$

where k_d (L mg^{-1}) represents the thermodynamic equilibrium constant; V (L) and m (g) are defined as the volume of the Ga^{3+} solution and the mass of the GO-AHZTA composite, respectively; T (K) represents the absolute temperature and R (8.314 J mol^{-1} K^{-1}) represents the ideal gas constant.

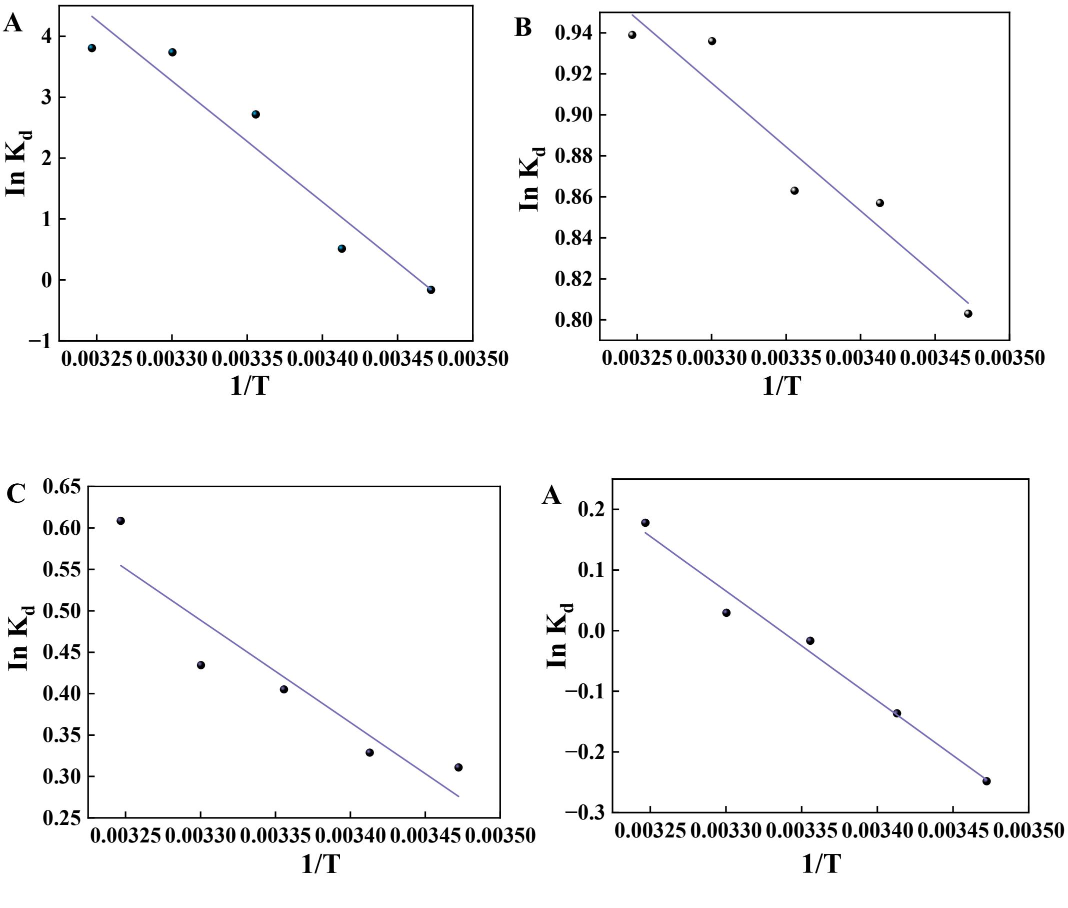

根

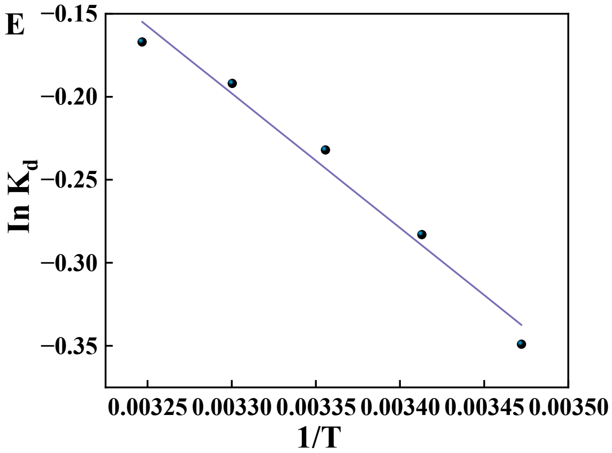

Fig. S2 Experimental data and the fitted curve of Ink$_d$ versus 1/T calculated from Van't Hoff plots of the GO-AHZTA composite for with different concentrations of Ga^{3+}: (A) 10 mg L^{-1}; (B) 20 mg L^{-1}; (C) 30 mg L^{-1}; (D) 40 mg L^{-1}; (E) 50 mg L^{-1}.

Table S1 Thermodynamic parameters of the adsorption by GO-AHZTA composite.

**C_0 of Ga^{3+} (mg L^{-1})**	***T* (K)**	$\Delta G^{\ominus}$ **(kJ mol^{-1})**	$\Delta H^{\ominus}$ **(kJ mol^{-1})**	$\Delta S^{\ominus}$ **(J mol^{-1} K^{-1})**
**10**	288	0.375	165.16	572.17
	293	-2.486		
	298	-5.347		
	303	-8.208		
	308	-11.068		
**20**	288	-1.932	5.19	24.72
	293	-2.056		
	298	-2.180		
	303	-2.303		
	308	-2.427		
**30**	288	-0.660	10.27	37.95
	293	-0.849		
	298	-1.039		
	303	-1.229		
	308	-1.419		
**40**	288	0.591	15.02	50.10
	293	0.341		
	298	0.090		
	303	-0.160		
	308	-0.411		
**50**	288	0.806	6.73	20.57
	293	0.703		
	298	0.600		
	303	0.456		
	308	0.394		

References:

[1] Ren, F.; Li, Z.; Tan, W. Z.; Liu, X. H.; Sun, Z. F.; Ren, P. G.; Yan, D. X., Facile preparation of 3D regenerated cellulose/graphene oxide composite aerogel with high-efficiency adsorption towards methylene blue. J Colloid Interface Sci 2018, 532, 58-67.

[2] M.M. Majd, V. Kordzadeh-Kermani, V. Ghalandari, A. Askari, M. Sillanpää, Adsorption isotherm models: A comprehensive and systematic review (2010− 2020), Science of The Total Environment, 812 (2022) 151334.
